# An Affordable Dual Purpose Spray Setup for Lithium-Ion Batteries Thin Film Electrode Deposition

**DOI:** 10.3390/ma17205114

**Published:** 2024-10-19

**Authors:** Dimitris Aivaliotis, Dimitra Vernardou

**Affiliations:** 1 Department of Electrical and Computer Engineering, School of Engineering, Hellenic Mediterranean University, 71410 Heraklion, Greece; 2 Institute of Emerging Technologies, Hellenic Mediterranean University Center, 71410 Heraklion, Greece

**Keywords:** lithium ion batteries, spray deposition setup, electrospray, air spray

## Abstract

This work presents a versatile and cost-effective spray setup that integrates both compressed air spray and electrospray techniques, specifically designed for small-scale laboratory use. This setup provides researchers with an accessible tool to explore spray methods for growing battery electrodes. While these techniques hold significant industrial promise, affordable and simple methods for their use in research settings have been limited. To address this, the setup includes custom control software and detailed information on costs and materials, offering an easy-to-implement solution. The system was tested with three samples per technique, using identical settings, to evaluate the repeatability of each method and gain insights into the uniformity and structure of the resulting films. The structural and morphological characteristics of the samples were analyzed using X-ray diffraction and scanning electron microscopy. The air-spray samples showed greater consistency and repeatability, whereas the electrospray samples exhibited better deposition results in terms of material coverage and higher crystallinity films. Cracking was observed in the air-spray samples, which was related to thermal stress, and both techniques exhibited solvent evaporation issues. The issues encountered with the setup and samples are summarized, along with possible solutions and the next steps for future upgrades and research.

## 1. Introduction

Lithium ion batteries (LIBs) are energy storage devices that store energy in the form of chemical energy and then release it into electrical energy. This process is completely reversible. Their operation is based on redox reactions, where a material gets oxidized, losing electrons and another material is reduced, gaining those electrons. These reactions happen between two electrodes, which are called active materials or working electrodes [1]. The positive (+) electrode of a battery is called cathode and the negative electrode (−) is called anode [1]. Energy conversion occurs through two processes: the discharge process, where an electric load is connected to two electrodes, and the charge process, where an external electric energy source with the same polarity, is connected [1].

LIBs have made significant advancements since their initial commercialization, with various materials being utilized as cathodes, starting with the stable lithium cobalt oxide (LCO), each offering distinct advantages and disadvantages [2,3]. One such material is lithium iron phosphate (LFP), introduced by John Goodenough and his team in 1997, belonging to the olive crystal structure group [4]. LFP offers the notable advantages of natural abundance, environmental friendliness, high chemical and thermal stability, high safety, and cyclability [3,5]. Although, it has lower capacity and potential compared to other cathode materials like TMOs, LFP’s lower potential [5] reduces side reactions with electrolytes, especially during large polarizations at high charging rates [5]. As an additional benefit, it is suitable for applications with aqueous-based electrolytes, which are known to be safer and less toxic compared to organic electrolytes [5].

In the following sections, we will explore the current manufacturing processes of LIBs, the key challenges facing the industry, and the proposed solutions. These sections provide a comprehensive overview of cutting-edge manufacturing techniques, pinpoint critical obstacles that need to be overcome, and detail how this work contributes to advancing the field with a versatile system capable of manufacturing both anodes and cathodes.

### 1.1. The Current LIB Manufacturing Process

**Figure 1:**
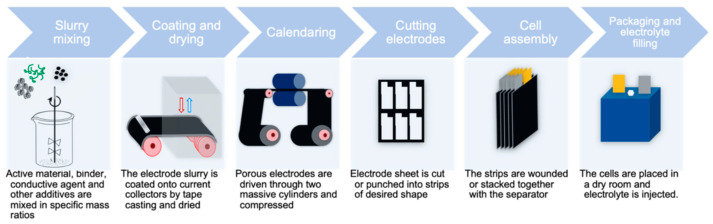
The six main manufacturing processing steps [7].

The industrial LIB cell production process has been standardized with a methodology referred to as powder technology [6]. The process can be categorized into six main steps: slurry mixing, coating and drying process, calendaring, cutting of the electrodes, cell assembly and packaging, and finally, electrolyte filling and cycling, as shown in Figure 1 [6,7,8,9]. In the first stage (slurry mixing process), the active material, like LFP, is mixed with a binder such as polyvinylidene fluoride (PVDF), a conductive additive (CA) like carbon black and a solvent, most commonly organic based like N-Methyl-2-pyrrolidone (NMP) [6,7,8,9], in specific ratios. This mixture, known as slurry, serves as the base for creating the working electrode of a battery cell after the mixing process [6,7,8,9]. The slurry is deposited onto the collection electrodes, which are usually Al or Cu, using a coating technique called slot die coating and a roll-to-roll (R2R) process [7,8,9]. The next step involves drying the electrode, where the solvent is evaporated, through convective air drying [6,7,8,9]. The electrodes are then compressed into a denser form, using one or two cylinders, also known as calendaring, which decreases the porosity, while increasing energy density and mechanical stability [6,7,8,9,10]. Sequentially, the electrodes are cut into desired shapes with either a chisel blade or laser cutting methods, with the necessary tabs for electrical connections formed during this process [8,9]. The electrodes are then stacked and welded together to form the final electrode, with appropriately sized separators placed between them for insulation [6,7,8,9]. Finally, the cells are assembled and filled with electrolyte in a dry room, a step referred to as the wetting process. This is followed by the formation and aging phases, where the cell undergoes cycling to form the solid electrolyte interface (SEI) layer and determine its final electrical characteristics before distribution [6,7,8,9].

### 1.2. Limitations of the Current Manufacturing Process

The powder technology, currently used for battery cell creation, is a resource-intensive and financially burdensome procedure. Environmental concerns have been raised due to the emission of greenhouse gases and material toxicity [6,7,8,9]. In industrial production, most of the problems arise from human resource dependencies and practical methods [9]. Effective coordination between production and electrical engineers is essential to reduce costs and optimize the balance between price and performance during the production and finishing stages of the cell [9]. The manufacturing process lacks well-defined parameters, and insufficient knowledge can lead to poor cell quality, over-engineering, high scrap rates, and costly test series [9].

Slot die coaters are known for their accuracy, controllability, and reliability, offering the notable advantages of high-speed coating uniformity in coating thickness. However, determining optimal parameters for the slurry composition used in slot die coating requires extensive trial-and-error, which can take up to three years and lead to significant material waste [9]. Additionally, setting parameters for a new formulation and establishing the relationship between the coating method and process parameters typically takes another month [9]. A common issue with slot die coaters is the presence of agglomerates, unwanted particles in the slurry that can block the coater, causing coating imperfections and high scrap costs [9].

Laser cutting, used for cutting electrodes and forming pole tabs, is highly effective due to its low heat input and precise cut quality. However, laser parameters must be adjusted according to the materials used, such as coated or uncoated Cu or Al foil [9]. Al foil usually contains a ceramic coating to enhance separator properties [11]. Occasionally, laser cutting may cause irregularities in the ceramic coating, leading to deviations in width and potential short circuits in the battery [9].

Cathode production accounts for up to 39% of the total energy consumption in electric vehicle battery creation [7]. This high energy use is primarily due to the drying process, which employs convective drying air and NMP as the solvent. NMP, with its high boiling point of 202 °C, requires a specialized solvent recovery system due to its high toxicity and flammability [7,8]. Despite these challenges, NMP remains popular in the industry for its high efficiency in dissolving PVDF, a critical component in the slurry composition, making it difficult to replace [6,7,8,9]. In contrast, distilled water has been successfully implemented for anode production, using carboxymethylcellulose (CMC) as the binder. Distilled water is non-toxic and has a lower boiling point of 100 °C, which reduces energy consumption [6,7,8]. However, its implementation for cathode production has not been successful due to issues such as poor wetting of the slurry, poor adhesion on metal current collectors, excessive secondary particle agglomeration in the slot die coater, Li leaching from the cathode due to corrosion, structural changes in aqueous cathode slurries and a more demanding drying process due to the highly reactive nature of water and its narrow voltage window [6,9].

The last notable problem is related to the aging process, which takes up to 25% of a factory floor space and takes 3–7 days for the wetting process and up to 2 weeks to complete [9,12]. During the initial cycles, the formation of the SEI layer consumes lithium mainly from the electrolyte, significantly decreasing the initial coulombic efficiency (ICE) of the cell [12]. To replenish the consumed electrolyte, it is refilled multiple times during the wetting stage [9,12].

### 1.3. Possible Solutions to Limitations and the Next Step in LIB Processing

One of the most promising solutions currently being explored by the industry is solid state batteries (SSB), which offer various advantages [7,9]. In SSBs, the electrolyte is in solid form, shortening the electrolyte filling step in the cell finishing process. Additionally, aging time can be reduced due to the faster SEI formation, and with a pure Li-metal anode, this step can be completely eliminated [9]. Alternatively, traditional liquid electrolyte filling can be combined with pre-lithiation technology, which provides lithium for SEI formation and assists in increasing ICE [9]. Pre-lithiation technologies are also important for enabling the usage of silicon alloys in anodes instead of traditional graphite, as silicon alloys are promising for next-generation batteries, but are hindered by severe volume changes during cycling, leading to poor ICE [9].

Regarding the solvent issue, the industry is exploring several solutions, including solvent reduction, alternative solvent recovery methods, water-based processing, and dry electrode processing [7,9]. Solvent reduction can decrease the amount of solvent used by up to 50% compared to traditional methods by replacing the slurry mixing technique with extrusion mixing or implementing curing technology [7]. Alternative solvent recovery methods involve mixing toxic solvents like NMP with another liquid, referred to as the washing agent, to facilitate evaporation [7]. Other solutions include replacing convective drying air methods with techniques such as near-infrared and laser drying [9]. 

Finally, alternative production methods are being explored, including solvent-free or water-based methods like electron beam curing, 3D printing, pulsed layer deposition (PLD), freeze casting/freeze drying, and dry pressed roll-to-roll manufacturing, and multiple spray-based techniques like spray printing, spray drying, and electrostatic spray deposition (ESD) [7,9]. Dry processing techniques are particularly attractive because they eliminate solvents, addressing both energy and toxicity issues [7,9]. Maxwell technologies have successfully implemented dry processing methods on a large scale, demonstrating their reliability [7,8,9].

### 1.4. The Advantages of the Spray Techniques and the Concept of This Research

Among the above techniques, the spray-based techniques have attracted significant attention due to their various advantages and method simplicity. These techniques are used for thin film electrode deposition, allowing multiple layers to be stacked through atomization using compressed air, a high DC voltage source, or both [7,13,14,15,16,17,18,19,20]. The primary advantages include eliminating electrochemically inactive materials in the slurry and offering flexibility in solvent selection, allowing the use of water or alcohol-based solvents instead of the toxic NMP. It is also possible to exclude solvent and proceed with a purely dry processing method [7,20]. Spray techniques enable the creation of very thin films down to the nanometer scale, through precise control of parameters such as liquid flow, air pressure, voltage, and substrate temperature. They are cost-effective, easily producible, and highly scalable [13,14,15,16,17,18,19,20]. ESD is the most promising candidate at the moment to replace the slot die coating technique in the industry, due to its high efficiency in transferring charged coating material, combined with thermal activation through a roll-to-roll process that takes only minutes [7,20].

Over the past decade, 3D printing technology has become widely accessible and reliable for consumers. Affordable and dependable basic 3-axes and Cartesian systems make excellent candidates for building spray setups. Following the aforementioned popularity of the two techniques in the current industry, we decided to develop a solution, which will encourage researchers worldwide to study the spray techniques and create new materials for the next generation of LIBs. Herein, we introduce a cost-effective, dual-purpose spray setup that employs both air-assisted and electrostatic spray deposition techniques for creating LIB electrodes. This setup was developed by modifying a standard low-cost commercial 3D printer and incorporating dedicated software to achieve high controllability and versatility, enabling the production of both anodes and cathodes. In this work, we discuss the challenges encountered during the development process, as well as the software options considered. Six samples of LFP cathode-three for each technique were produced to initially validate the system. These tests evaluated the setup’s deposition capabilities and the repeatability of each method. The results highlighted the importance of precisely tuning solution parameters, such as surface tension, along with key settings like volumetric flow rate and temperature, to achieve optimal deposition in both techniques.

## 2. Methods and Materials

### 2.1. Construction of the Spray System and Crucial Software Features

**Figure 2:**
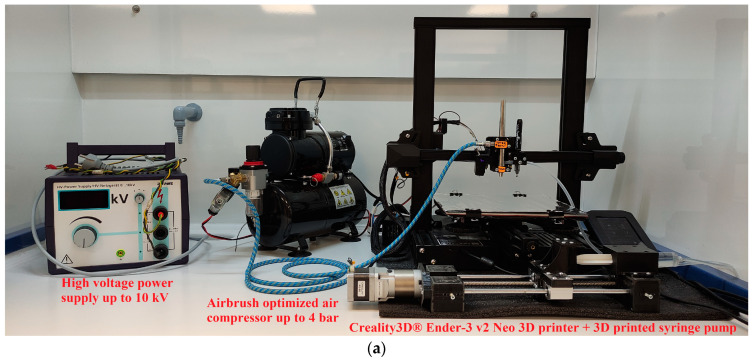

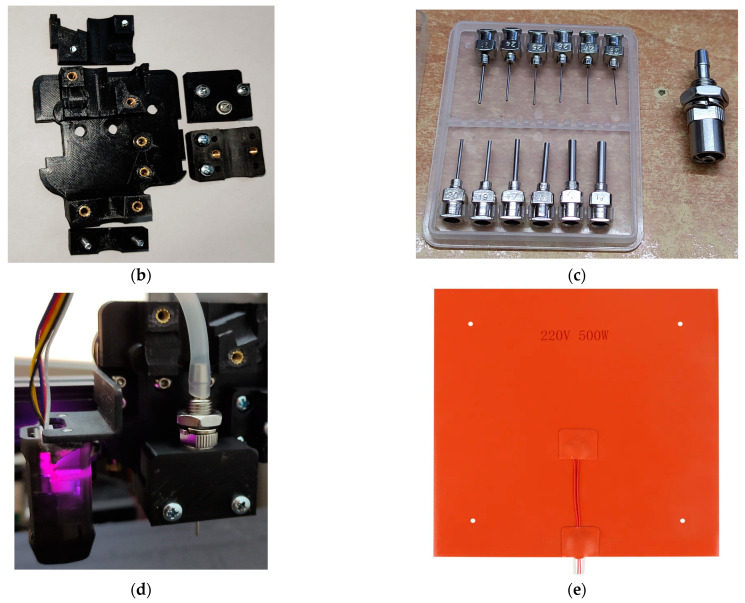
(**a**) The setup with all the utilized equipment, including a high voltage power supply, an air compressor, the 3D printer and a 3D printed syringe pump; (**b**) The 3D printed adapter used to hold the spray gun on the printer; (**c**) The luer to bar adapter with its set of needles; (**d**) The luer to barb adapter placed into its 3D printed adapter and attached to the spray gun adapter; (**e**) The silicon mains powered heater upgrade for the hotplate [22].

An Ender-3 V2 Neo 3D printer (by Shenzhen Creality 3D Technology Co., Ltd., Shenzhen, China 518131) was selected for conversion due to its popularity, reliability, and low cost, which can be observed in Figure 2a. A custom, professionally tuned version of the open-source Marlin firmware, provided by Miguel Risco-Castillo (also known as MRiscoC) on the Github page (Marlin Version 2.1.3 MRiscoC), was installed on the printer [21]. The printer was converted into a spray setup through the design of a 3D printed adapter in a CAD program, which was placed upon the X-carriage (*X*-axis carrier) of the printer as shown in Figure 2b. The adapter was designed to support the placement of a Ultra two-in-one series airbrush (by Harder & Steenbeck Gmbh & Co. KG, Norderstedt, Germany), which acted as the spray gun of the setup aligning the nozzle with the printer’s original hotend nozzle. The electrospray function required a capillary (needle) based system to operate [19]. A stainless steel luer (male) to barb (4.7 mm OD) adapter was selected and paired with a set of stainless steel needles, providing a variety of useful capillary needle diameters for the setup as shown in Figure 2c. Another 3D-printed adapter was designed to support the placement of the luer-to-barb adapter onto the existing spray gun adapter, as presented in Figure 2d. A highly flexible silicon tube 3 × 5 mm (ID–OD) was selected as the feeding tube for the spray gun and the luer-to-barb adapter due to its affordability, excellent flexibility, and high corrosion resistance.

During initial testing, accurately positioning the substrate (electrode) on the stage surface proved challenging. To address this, a marking pen was used to draw the substrate outlines for proper placement. An adapter for the pen was designed, 3D printed, and positioned next to the spray gun to minimize offsets from the center of the spray gun nozzle and avoid wasting space on the setup surface. Although the silicon tube fit perfectly into the spray gun’s input feeding hole, it detached during tests due to the machine’s movements. To prevent this, a small 3D printed adapter was placed next to the feeding hole, on top of the marker pen adapter, securing the silicon tube with three screws. Another significant issue was the inadequate evaporation of the solvent, as the printer’s hotplate (heated bed) could not reach the temperatures fast enough nor maintain them due to the cooling from the incoming compressed air, originating from the spray gun. To resolve this, the printer’s hotplate was upgraded with a 500 W, 230 V (mains voltage) silicon heater (Figure 2e [22]) and cotton insulation to further assist in temperature maintenance. The silicon heater’s power was controlled through a solid-state relay (SSR – model ASR-15DA by ANLY electronics Co., Ltd., Taipei, Taiwan [23]), using the original 24 V hotplate supply as a control signal. To ensure safety, the SSR and mains voltage were enclosed in an isolation box, which was designed, 3D printed, and placed on the side of the printer. Additional equipment includes an airbrush-optimized air compressor, model AS-186 (Intertek Group plc, 33 Cavendish Square, London, England W1G 0PS) and a high voltage power supply up to 10 kV (by PHYWE Systeme GmbH & Co. KG, Göttingen, Germany), shown in Figure 2a. The compressed air supply to the spray gun is controlled through a 24 V solenoid air valve using the repurposed fan control pin, which is in turn controlled by the printer through software.

To achieve a uniform coating on the electrode surface and control the amount of solution deposited in a single sprayed layer, a stable and precise solution-feeding method is essential. Syringe pumps, which deliver a precise amount of solution with a steady flow by pushing the plunger of a syringe at a constant velocity, are commonly used in laboratory experiments. However, even the more affordable commercial syringe pumps are typically expensive. The advent of 3D printing technology has enabled the design and fabrication of custom parts, including laboratory instruments, providing cost-effective and reliable alternatives [24,25,26,27,28]. In this paper, a 3D-printed syringe pump was designed, based on this concept, as illustrated at the front of the printer in Figure 2a. Analytical information about the structure and issues faced with the syringe pump, along with costs for the setup and the syringe pump are provided in the Supplemental Material through Tables S1 and S2 and Figures S1 and S2.

### 2.2. The Dedicated Software and Its Most Important Features

**Figure 3:**
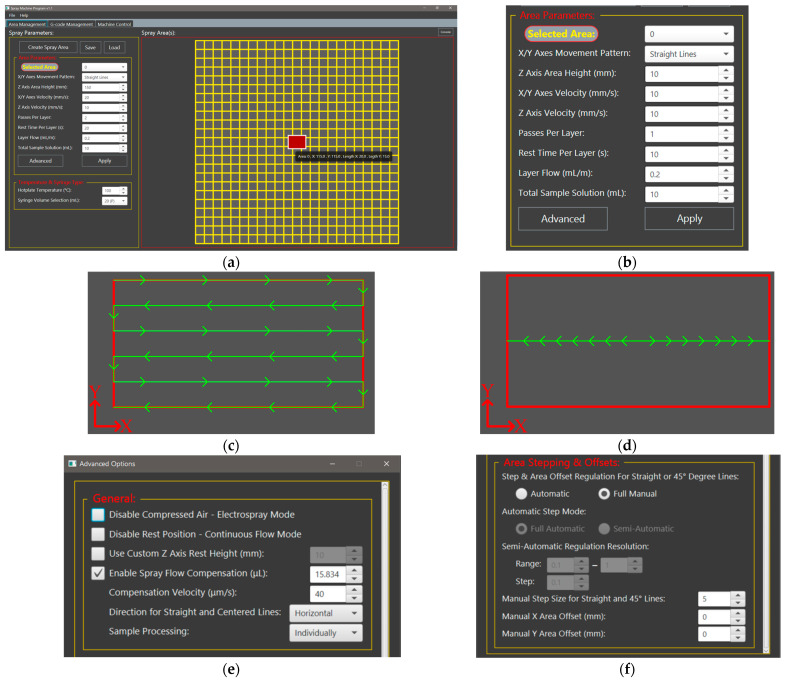
(**a**) The “Area Management Tab”, handling the sample placement and main spray settings for the process; (**b**) The main settings used for the spray process of a sample; Movement pattern example for the (**c**) “Straight Lines”; and (**d**) “Centered Line(s)” pattern; The advanced options window: (**e**) “General” options; (**f**) “Area Stepping & Offsets” options.

The program was designed with a straightforward concept, where deposition occurs in layers, allowing the solvent from the previous layer to evaporate completely before spraying the next layer. Users also have the option to switch to a continuous flow operation, bypassing the layered structure process entirely. Given that the printer uses Marlin firmware, G-code is the primary control language. Therefore, the program was designed to generate optimized G-code for the spray processes. The interface is organized into three tabs: “Area Management”, “G-code Management”, and “Machine Control”. The first tab, which is the most important tab, involves the creation of a spray project, based on an interface that utilizes hotplate dimensions of 230 × 230 mm (mm), through the MRiscoC firmware (stock dimensions of 220 × 220 mm) [21]. This interface allows for the placement of rectangular-shaped areas onto the hotplate based on the X and Y axes coordinates. An example of this interface is shown in Figure 3a. A spray area is created using the “Create Spray Area” button, which opens a new window, prompting the user to input the placement coordinates and size of the area. On the left side of the tab, users can set specific parameters for the selected area. The main settings available are: “X/Y Axes Movement Pattern”, “Z Axis Area Height (mm)”, “X/Y Axes Velocity (mm/s)”, “Z Axis Velocity (mm/s)”, “Passes Per Layer”, “Rest Time Per Layer (s)”, “Layer Flow (mL/m)”, and “Total Sample Solution (mL)”. These settings can be applied to an area using the “Apply” button located below and can be better observed in Figure 3b. The “G-code Management” and “Machine Control” tabs in the software allow for viewing and editing G-code as needed, as well as direct control and interaction with the setup through serial communication. Additional information can be found in the Supplementary Material and Figure S3a,b. The software was designed exclusively by us, and the utilized version for our tests was v1.1. 

The “X/Y Axes Movement Pattern” parameter offers four preprogrammed spray patterns: “Straight Lines”, “45° Angled Lines”, “Centered Line(s)”, and “Stationary Center”. Two of these patterns were selected and used in the experimental section. For the “Straight Lines” pattern, the spray gun starts at the top left corner of the substrate, moving in a straight line to the opposite end. It then makes a small perpendicular step and repeats the straight-line motion to the other end, covering the entire surface with evenly spaced lines. The “Centered Line(s)” pattern starts with the spray gun positioned at the center of the substrate width, moving in a straight line to the opposite end and then returning back to the starting point. Users can select the number of repetitions for this pattern, which is suitable for small samples. Examples of the “Straight Lines” and “Centered Line(s)” patterns are depicted in Figure 3c,d and additional information about the “45° Angled Lines” and “Stationary Center” patterns is provided in the Supplementary Material and Figure S4.

The “Z Axis Area Height (mm)” parameter defines the height of the *Z*-axis during the spraying process, which influences the spray cone diameter and the degree of overlap above the substrate in air-assisted spraying, as well as affecting operation mode and voltage regulation in electrospray applications [29]. The “X/Y Axes Velocity (mm/s)” controls the movement speed of the X and Y axes during spraying; lower velocities result in greater material deposition per layer, while higher velocities lead to reduced deposition. The “Z Axis Velocity (mm/s)” sets the speed of *Z*-axis movement, although this is generally less critical compared to the X/Y axis velocity. The “Passes Per Layer” parameter defines how many times the selected spray pattern is applied per layer, and for the “Centered Line(s)” pattern, it specifies the number of lines sprayed in each layer. The “Rest Time Per Layer (s)” determines the waiting period after completing a layer before initiating the next one. A key parameter, “Layer Flow (mL/m)”, establishes the syringe pump flow rate during pattern movement, which remains constant throughout the process. The “Total Sample Solution (mL)” indicates the total volume of solution applied to the substrate, while the “Hotplate Temperature (°C)” allows users to set the desired temperature of the hotplate. Users can also select the syringe type and its corresponding volume for use in the pump, with four standard options available: a 10 mL and a 30 mL glass syringe, as well as 20 mL and 30 mL single-use plastic syringes. In the experimental section, the 20 mL single-use plastic syringes were utilized.

The “Advanced” button opens a new window with additional critical options for the spray processes, as shown in Figure 3e,f. For the experimental section, key settings in the “General” section include:

- “Disable Compressed Air–Electrospray Mode”: This option completely disables the compressed air, switching the system to electrospray operation.
- “Use Custom Z Axis Rest Height (mm)”: This allows to set the *Z*-axis to a specific position during the waiting period between layers. This is particularly useful in electrospray mode, as it disrupts the electric field, effectively pausing the electrospray function, along with the cutoff of solution supply from the syringe pump.
- “Enable Spray Flow Compensation (μL)”: This function addresses the issue of initial solution shortage by pushing a small amount of solution at the start of each layer. The syringe pump velocity can be adjusted using the “Compensation Velocity (μm/s)”. This is crucial as the spray gun or capillary needle often fails to spray at the beginning of each layer due to insufficient liquid, and adjustments are made based on the liquid properties and spraying technique.

In the “Area Stepping & Offsets” section, specialized settings for the “Straight Lines” and “45° Angled Lines” patterns are available, related to the small step performed before each line. The “Automatic” option uses algorithms to determine the step size based on the substrate dimensions, while the “Full Manual” mode, used in the following experiments, allows users to manually set the step size. Additional information about non-critical features for this work is provided in the Supplementary Materials.

### 2.3. Setup Evaluation, Growth Parameters, and Characterization of LiFePO_4_

The solution consisted of 40 mL of deionized water (H_2_O), 40 mg of commercial carbon-coated lithium iron phosphate (LFP/C—purchased from Sigma Aldrich, Athens, Greece), and 40 mg of polyvinylpyrrolidone (PVP—purchased from Sigma Aldrich, Athens, Greece) in a 1:1:1 ratio, with PVP serving as a binder. This mixture underwent sonication for 30 min. Due to the non-conductive nature of the initial solution, it was unsuitable for the electrospray technique, which requires a conductive liquid [29]. To achieve the necessary conductivity, a few drops of a 1M lithium chloride (LiCl—Sigma Aldrich, Athens, Greece) solution in deionized water were added, adjusting the conductivity to approximately 1500 μS/cm from about 35–40 μS/cm. During the tests, the printer’s hotplate was covered with Al foil, and the current collectors used were pure, cleaned Al metal with dimensions of 20 × 9 mm (±1 mm) for the deposition of LFP on the top of them. Additionally, the Al foil served as the grounded collector, with an alligator clip connected to a negative voltage source and the positive voltage applied to the luer-to-barb adapter, which was paired with a 0.25 mm needle (26G). The specific settings chosen for each technique, which were optimized based on the properties of the solution and the technique, are detailed in Table 1. Characterization of the samples included X-ray diffraction (XRD) results, using a SmartLab^®^ SE (by Rigaku Europe SE-Hugenottenallee 167 Neu-Isenburg 63263, Germany) instrument, with the following processing parameters: power 46 kV, 50 mA, step 0.01° and speed time 8°/min, angle range: 5°–80° and wavelength type Ka-0.15406 nm (1.540593 Å) and 0.15444 nm (1.544414 Å). Additionally, field-emission scanning electron microscopy (FE-SEM) was used to investigate the morphology of the samples, using a JSM-IT700HR InTouchScope™ Field Emission SEM (by Thermo Fisher Scientific-Neuhofstrasse11, 4153 Reinach TechCenter, 4153 Basel, Switzerland), along with the following processing parameters: 15 kV power, 10 μm width.

## 3. Results and Discussion

The following paragraphs evaluate the uniformity and repeatability of the processes, both of which are crucial for ensuring consistent performance. These factors play a vital role in enhancing safety by reducing the risk of hot spots and localized overheating, while also ensuring reliable outcomes through consistent electrode characteristics, leading to predictable and stable results.

**Figure 4:**
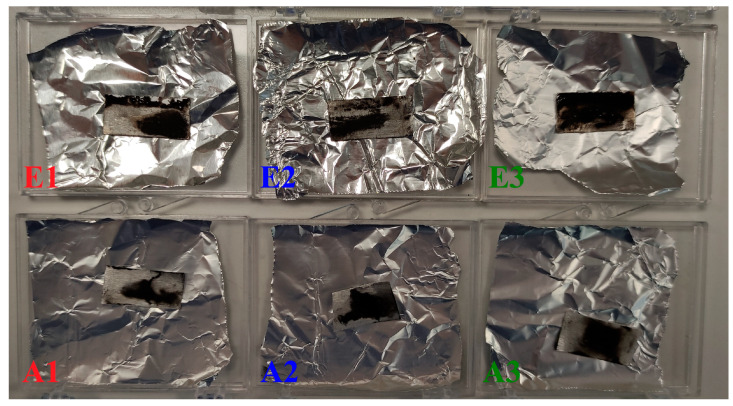
The six produced spray technique samples, three electrospray samples (**E1**,**E2**,**E3**) on top and three air-assisted spray samples on bottom (**A1**,**A2**,**A3**).

The six sprayed samples are displayed in Figure 4, where the top three samples (Electrospray 1—E1, Electrospray 2—E2, and Electrospray 3—E3) are from the electrospray method, and the bottom three samples (Air-Spray 1—A1, Air-Spray 2—A2, Air-Spray 3—A3) are from the air-assisted spray method. It is evident that all samples show poor coverage of the sprayed material on the surface. This poor coverage may be attributed to the properties of the solution, particularly its surface tension, as the solvent formed a large drop on the Al surface after the completion of each layer in both techniques [30]. To evaluate the repeatability of each technique, the mass of all substrates was measured with a precision scale before and after the deposition process. The air-assisted spray samples consistently had a deposited mass close to 2 mg, whereas the electrospray samples showed varying results with deposited masses of around 5 mg (E1), 3 mg (E2), and 14 mg (E3). The large variations of the electrospray mass samples are most likely related to the high voltage power supply, since all the other parameters were the same and it was the only value fluctuating during the process, about ±0.4 kV, due to inherently poor voltage regulation.

**Figure 5:**
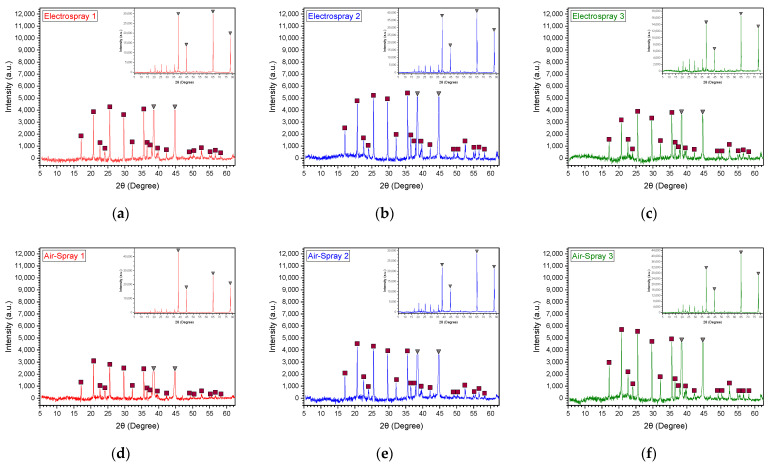
XRD patterns of: (**a**) E1; (**b**) E2; (**c**) E3; (**d**) A1; (**e**) A2; (**f**) A3; Peaks of LFP/C and Al are indicated with square and triangle respectively.

In Figure 5, all XRD patterns for each sample are shown. Each graph is focused on the area of interest for the deposited material, but an inset graph with the original measurement is provided in the top right corner. The four large peaks in the original graph belong to the Al substrate, which was verified using a reference pattern of pure Al from [31]. The two remaining 2θ peaks around 38.4 and 44.6 degrees were cropped out to provide better insight into the materials of interest. Using the JCPDS No. 40-1499 reference pattern, which was acquired from [32], almost all the carbon-coated LFP composite (LFP/C) peaks were verified. The PVP binder, being an amorphous material, displays an increasing intensity pattern between approximately 60 and 20 degrees, followed by a decline down to 10 degrees, as observed in the pure PVP pattern data from [33]. This broad feature could obscure the distinct peaks of the active material and was therefore removed during data processing to enhance clarity. The sharp peaks indicate a good crystallinity of LFP/C in all samples. However, the peak intensity of the electrospray samples is higher than that of the air-assisted samples, suggesting that electrospray can produce high crystallinity layers due to the increased deposited mass. Finally, the variation in peak intensity among samples may be attributed to differences in sample thickness, as the Al is not uniformly covered in all cases independently of the spraying process.

**Figure 6:**
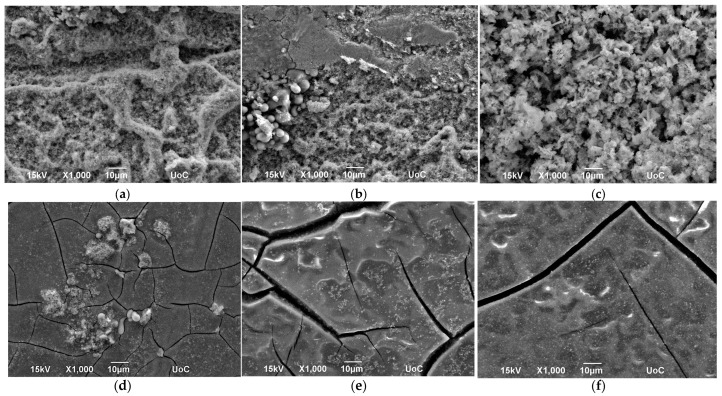
SEM images of: (**a**) E1; (**b**) E2; (**c**) E3; (**d**) A1; (**e**) A2; (**f**) A3.

Figure 6 presents SEM images of all samples. Despite the differing outcomes between the two techniques, the three samples from each technique exhibit a highly similar morphology. The electrospray samples demonstrate significant particle agglomeration, whereas the air-assisted spray samples possess a smooth surface finish with prominent large cracks. These cracks likely result from thermal stresses and the rapid deposition of layers, as the flow rate for the air-assisted spray was set at 1.5 mL/min, with deposition conducted over approximately 1 h and 50 min [34]. The hotplate temperature for the air-assisted spray was set at 120 °C, which is lower than that for the electrospray because the compressed air from the spray gun facilitated the evaporation process and the solution was dispersed over a large spray cone. However, the rapid surface cooling and heating likely induced stress on the substrate, leading to the formation of cracks. In contrast, the electrospray flow rate was set at 0.25 mL/min, enabling a more even and gradual deposition that did not show cracks, except for some visible cracks in sample E2. This method required nearly 5 h. Notably, the electrospray operated in cone-jet mode [29], which provided excellent substrate coverage with the “Straight Lines” pattern, resulting in less material waste compared to the air-assisted spray.

Overall, deposition was successfully achieved using both techniques, despite suboptimal results. This confirms that the spray setup is functional, which was the primary objective of this work, rather than an exhaustive search for optimal parameters and samples. The test conditions were not fully optimized; for example, the properties of the solutions, such as viscosity and surface tension, were not adjusted, and the spray parameters could benefit from further refinement. Significant differences in flow rates and hotplate temperatures between the two techniques were primarily influenced by both the nature of the technique and the solution properties. For the air-assisted spray, flow rates below 1.0 mL/min resulted in completely unstable spray flow, and rates under 1.2 mL/min led to inconsistent performance. A flow rate of 1.5 mL/min induced rapid deposition with a substantial amount of solution per layer, necessitating longer rest times for each layer and higher hotplate temperatures to ensure sufficient evaporation. This led to thermal stress and cracking due to rapid cooling from the compressed air, as previously discussed. In contrast, for the electrospray technique, flow rates above 0.25 mL/min resulted in insufficient solvent evaporation due to the large amount of solution deposited per layer. This required excessive hotplate temperatures to facilitate evaporation, as there was no airflow source except for the fume extractor under which the setup was placed. Additionally, the selected flow rate, combined with the 20 mL solution, resulted in an impractical deposition time of nearly 5 h, making it unsuitable for realistic applications. Although the focused beam in cone jet mode and the slower deposition resulted in increased material deposition, the amount deposited varied significantly among the samples, likely due to voltage fluctuations during the process.

A possible solution to mitigate these cracks, which appear in all air-assisted samples, could include slowing down the deposition process by reducing the flow rates. However, in this instance, this approach was not feasible due to the properties of the solutions, which resulted in inconsistent performance at lower flow rates. Another option would be to increase the X and Y axes velocities during deposition, which would inherently decrease the amount of material deposited per layer while maintaining a constant flow. Additionally, the choice of a high hotplate temperature introduced thermal stress to the substrate, in combination with the incoming compressed air from the spray gun. In this particular case, it was impossible to select a lower temperature due to the solvent evaporation issues, meaning that the properties of the solution were once again the limiting factor. While water is less energy-intensive as a solvent compared to NMP, usually used in industrial and laboratory applications, it remains a relatively energy-demanding solvent. Therefore, replacing water partially or entirely with a lower energy-demanding solvent, such as ethanol, could enable the use of higher flow rates at lower temperatures, or ideally, achieve a reduction in both flow rate and temperature. Adjusting the air pressure from the air compressor could also make a significant impact, as it directly affects the velocity of air hitting the substrate and its cooling effect. Finally, the total amount of solution, being 20 mL in this case, was an important indirect limiting factor due to the impractical deposition time, especially during the electrospray process. Future experiments with decreased solution quantity, improved solution properties, and settings selection may lead to superior sample fabrication.

The overall cost of the setup is reasonable, with the high-voltage power supply being the most expensive component, and it is possible to design a low-cost option that could significantly reduce the total cost [35]. A direct comparison between this setup and industrial solutions presents challenges due to the reliance on price quotations rather than standardized pricing from manufacturers. Furthermore, performance comparisons are not suitable given the substantial differences in production quality and the advanced engineering involved in developing those systems. This setup is specifically designed for small-scale laboratory applications, providing researchers worldwide with a practical tool to initiate studies on new materials development using spray techniques. Designing a 3D printing system from scratch can be expensive, as it requires purchasing individual components. In contrast, pre-assembled printers are more affordable, thanks to manufacturers’ ability to source materials at reduced prices. Furthermore, choosing a commercially available 3D printer not only reduces costs, but also saves users the time and effort required in building, programming, and calibrating the system.

Notably, the setup retains potential for scale-up and industrial applications by focusing on fundamental and critical parameters, such as three-dimensional position control, which simulates roll-2-roll processes and selectable fixed spraying distances, adjustable, stable low and high flow rates of liquid into the spray gun or capillary needles for small and large samples, along with controllable air pressure and high voltage. Additional features, like preprogrammed movement patterns, provide valuable reference points and enhance repeatability, as demonstrated in the air-assisted samples when solution parameters are optimized and standardized.

The setup offers high customizability through its software, which can be further improved in the future. Its modifications have been relatively simple, adding to its versatility. Moreover, the syringe pump provides high precision in its movements and solution delivery, synchronized with the printer’s main axes through the extruder’s stepper driver rather than an external controller. Insufficient evaporation was observed in both techniques, likely due to the challenges of evaporating water, which were discussed earlier. A crucial future upgrade would be an evaporation assistance system, such as the conventional convective drying air used in the industry, or emerging options like near-infrared and laser drying, to assist in the evaporation of more demanding solvents. The spraying solution could be further optimized by new solvent mixtures, which are less energy demanding compared to pure water, along with surfactants to tune surface tension and possibly the viscosity [36], while maintaining reasonable evaporation properties for the solution.

## 4. Conclusions

This cost-effective, dual-purpose spray setup achieved its primary goal by successfully producing six electrodes, three using each technique, and demonstrating effective deposition capabilities. The air-assisted spray technique delivered more consistent and repeatable results, while the electrospray technique showed promise for achieving higher crystallinity layers. Although the modifications to the setup were straightforward and budget-friendly, the high-voltage power supply was a notable exception, with the potential for substitution with more affordable alternatives. Additionally, sourcing materials from economical suppliers like Aliexpress could further reduce costs. The included software offers essential yet customizable functions for both techniques, with opportunities for future enhancements. This setup marks a significant initial step for researchers exploring spray techniques. However, improvements are needed, particularly in managing solvent evaporation, which could be addressed by incorporating an evaporation assistance system. Future efforts should focus on optimizing spraying solutions and parameters to refine electrode performance through electrochemical evaluation studies. Importantly, this versatile setup holds potential for adaptation to other applications beyond lithium-ion battery electrodes.

## Figures and Tables

**Table 1:** The selected settings for each technique.

Setting	Air-Assisted Spray	Electrospray
X/Y Axes Movement Pattern	Centered Line(s)	Straight Lines
Z Axis Area Height (mm)	150	12.5
X/Y Axes Velocity (mm/s)	20	20
Z Axis Velocity (mm/s)	10	10
Passes Per Layer	6	1
Rest Time Per Layer (s)	45	25
Layer Flow (mL/m)	1.5	0.25
Total Sample Solution (mL)	20	20
Hotplate Temperature (°C)	120	170
Disable Compressed Air–Electrospray Mode	No	Yes
Use Custom Z Axis Rest Height (mm)	No	30
Enable Spray Flow Compensation (μL)	15.834	6
Compensation Velocity (μm/s)	40	40
Area Stepping (mm)	Not applicable	1.5 (Full Manual)
Air pressure or High Voltage	1 bar	8 kV (±4)

## Data Availability

The original contributions presented in the study are included in the article/Supplementary Materials, further inquiries can be directed to the corresponding authors.

## Supplementary Materials

The following supporting information can be downloaded at: https://www.mdpi.com/article/10.3390/ma17205114/s1, Figure S1: The designed syringe pump from various viewing angles; Figure S2: (a) Top view of the syringe adapters; (b) Side view of the syringe adapters; (c) A glass syringe with its flexible plunger mechanism to prevent destruction; (d) The flexible mechanism of the plunger up close; Figure S3: (a) The “G-code Management” tab with a generated example; (b) The “Machine Control” tab, used to communicate directly with the setup; Figure S4: Movement pattern example for the “45° Angled Lines” pattern; Figure S5: The “Advanced Options” window (a) “General” options; (b) “Area Stepping & Offsets” options; and Vertical operation for (c) The “Straight Lines” pattern; (d) The Centered Line(s) pattern; Table S1: The analytical cost of the syringe pump, along with the materials procurement methods; Table S2: The analytical and total cost of the spray setup; Table S3: The cost of the syringe pump compared to other available options; Table S4: The “Full Automatic” searching step algorithm.
