# Supplementary material for: An Affordable Dual Purpose Spray Setup for Lithium-Ion Batteries Thin Film Electrode Deposition
Source: Materials (Basel). 2024 Oct 19;17(20):5114. doi: 10.3390/ma17205114 (PMC11509203; doi:10.3390/ma17205114)
Supplement: Supplementary file 1 [file materials-17-05114-s001.zip › materials-3242282-supplementary.pdf]

materials

MDPI

Supplementary material

An Affordable Dual Purpose Spray Setup for Lithium-Ion Batteries Thin Film Electrode Deposition

1. The 3D printed syringe pump and analytical costs for the setup

The construction of the 3D printed syringe pump, which was initially designed, is shown in Figure S1. Two 8 mm linear rods are secured at each end by two 3D printed bases. Two aluminium bases, each with an 8 mm linear bearing, are mounted onto the rods and connected by a third 3D printed base. Motion within the system is facilitated by an 8 mm lead screw paired with an anti-backlash T-nut, which is inserted into the 3D printed base joining the two aluminum bases. One end of the lead screw is supported by an 8 mm pillow block and a concealed 8 mm bearing on each end. The opposite end of the lead screw is connected to an 8-8 mm rigid shaft coupler, which is attached to a 100:1 precision planetary gearbox from StepperOnline® (model EG17-G100). The gearbox is driven by a repurposed Nema17 stepper motor from the printer's extruder (Creality 42-40). Most of the screwing holes of the syringe pump were reinforced with M3 threaded inserts, along with two M5 inserts for the pillow block to prevent mechanical wear.

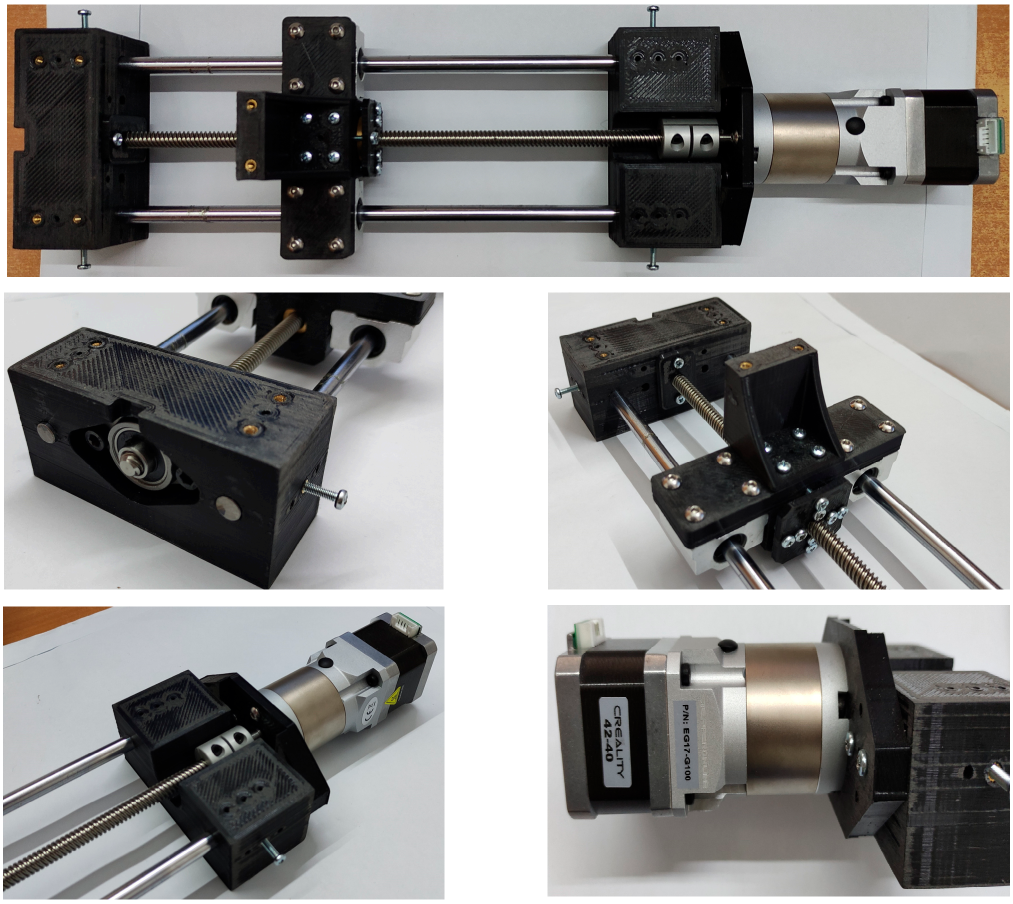

Figure S1. The designed syringe pump from various viewing angles.

The syringe pump incorporates a 3D-printed component, which is secured to the base connecting two aluminum platforms, designed to actuate the syringe plunger. Four types of syringes were selected for use with the syringe pump: two glass syringes of 10 mL and 30 mL, and two single-use plastic syringes of 20 mL and 30 mL. Glass syringes provide advantages such as minimal chemical reactivity and high corrosion resistance, but are significantly fragile. Notably, the specific glass syringes were sourced from a low-cost Chinese supplier and exhibited leakage at the plunger's rear due to insufficient quality control. Plastic syringes, while more flexible and less prone to breakage, suffer from imprecise movements due to excessive flexibility. Despite the drawbacks of glass syringes, they were retained for future experiments involving highly corrosive chemicals. In contrast, all practical experiments were conducted with plastic syringes, as they were suitable for the non-corrosive chemicals used.

Each syringe was equipped with a custom-designed adapter for the syringe pump, as illustrated in Figures S2a and S2b, arranged from left to right as follows: 10 mL glass, 20 mL plastic, 30 mL plastic, and 30 mL glass. While standard syringe pumps typically use a universal syringe holding adapter [1], four distinct adapters were designed to provide a better grip on the syringes. Additionally, each syringe had a corresponding plunger holder, depicted in Figures S2a and S2b, positioned in front of the adapters. These holders secured the syringe plunger to the base that actuates it, using a flexible mechanism shown in Figures S2c and S2d to prevent damage to glass syringe plungers. All the used materials for syringe pump, along with their costs, are displayed in Table S1, while overall cost of spray setup is displayed in Table S2. The subsequent Table S3 compares the cost of the syringe pump to various other open source options, including commercial options. As can be observed in the prices, the commercial syringe pumps are highly expensive, even the lower specification models. On the other hand, the open source options are performing sufficiently for their design and price. A notable advantage of an open source pump syringe is the customization, where features can be added depending on the application. STL files for the syringe pump and the printer modifications will be provided under request and direct communication with the main author of this project.

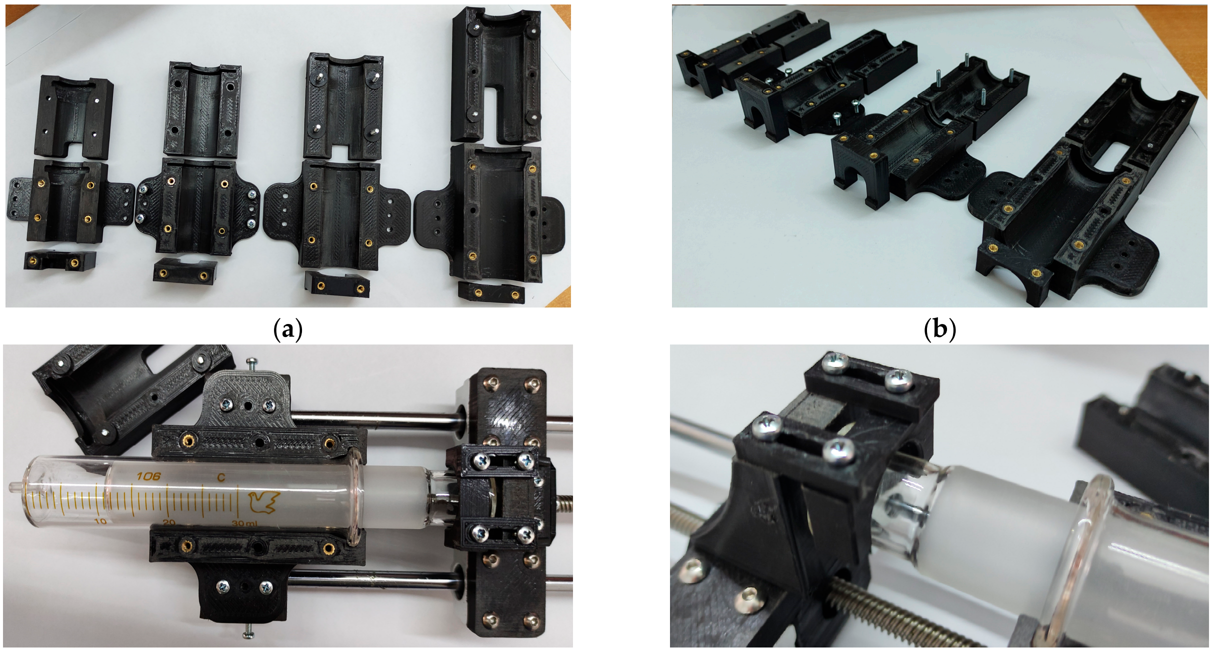

(a) (b) (c) (d)

Figure S2. (**a**) Top view of the syringe adapters; (**b**) Side view of the syringe adapters; **(c)** A glass syringe with its flexible plunger mechanism to prevent destruction; **(d)** The flexible mechanism of the plunger up close.

Table S1. The analytical cost of the syringe pump, along with the materials procurement methods.

Mechanical Part(s).	Quantity	Obtained method	Total Part(s) Cost (€)*
Linear Rod 8 mm OD – 300 mm Length	2	Purchased	6
Linear Ball Bearing Block 8 mm (Model –SC8UU)	2	Purchased	7,6
T8 Lead Screw 8 MM Lead OD – 300 mm Length	1	Purchased	5.9
T8 Anti-Backlash T-Nut for Lead Screw	1	Purchased	2.9
Pillow Block 8 mm (Model – KFL08)	1	Purchased	2.2
Flanged Ball Bearing (Model – MF128ZZ)	1	Purchased	1.4
8 to 8 mm Rigid Shaft Coupler	1	Purchased	2.55
M3 Threaded Insert	18	Purchased	0.97
M5 Threaded Insert	2	Purchased	0.6
Planetary Gearbox 100:1	1	Purchased	56.74
Main Base part 1 (Motor Side)	1	3D Printed	2.7
Main Base Part 2 (Syringe Side)	1	3D Printed	2.7
Plunger Holder Support Base	1	3D Printed	1.17
Plunger Holder	1	3D Printed	0.4
Gearbox Holding Adapter	1	3D Printed	0.6
Bearing Cover	1	3D Printed	0.06
T-Nut Extra Support Cover	1	3D Printed	0.08
**Total Cost**			**94.57€**

*3D printed parts cost is calculated using eSUN® plastic price/kg around the time it was purchased.

Table S2. The analytical and total cost of the spray setup.

Component.	Supplier	Total Cost (€)
Creality3D® Ender-3 V2 Neo 3D printer	Local (Greek)	261.2
Pump Syringe – 3D Printed Parts Excluded	Various	86.86
24V Solenoid Air Valve 8 bar max – G1/8 Female to Female	Local (Greek)	16.25
G1/8 to G1/8 Male to Male converter	Local (Greek)	5
1 Kg Black eSUN® PLA+	Local (Greek)	19.2
Anly® Solid State Relay – Model ASR-15DA	Local (Greek)	17.57
Silicon Heater – 500W 230V	Aliexpress	15.59
Cotton Insulation	Aliexpress	1.9
Luer Male to Barb 4.7mm Stainless Steel Adapter	Aliexpress	18.92
Set of 12 Luer Type Stainless Steel Needles	Aliexpress	3.83
Glass Syringe 30 mL	Aliexpress	7.05
Glass Syringe 10 mL	Aliexpress	6.2
Plastic Single-use Syringes 20 mL – Box of 50	Local (Greek)	9.92
Plastic Single-use Syringes 30 mL – Box of 40	Local (Greek)	10.42
Black Marker Pen	Local (Greek)	2.8
High voltage power supply – 10 kV	Pre-Existing*	1355.9**
Airbrush (spray gun)	Pre-Existing*	127
Air compressor for the airbrush	Pre-Existing*	139.9
**Total Spray Machine Project Cost (Roughly)**		**2156.49**

*The equipment existed in the laboratory from previous experiments.
**Price acquired from Phywe official Europe online store and can vary depending on the situation.

Table S3. The cost of the syringe pump compared to other available options.

Instrument Availability	Manufacturer (and Model)	Cost (€)	Reference
Under request*	D. Aivaliotis	94.57 €	This work
Open source	A. S. Samokhin	<100.0	1
Open source	Various	49.29	2
Open source	Various	87.49	3
Commercial	KF TechnologyNE-300	499.0	4
Commercial	World Precision InstrumentsAL-1000	987.0	5
Commercial	OssilaL2003S2	1380.0	6
Commercial	DK InfusetekISPLab01	1298.4	7

*STL files will be granted under request from the corresponding manufacturer

2. A closer look into the dedicated software

The tab named “G-code Management”, displayed in Figure S3a is used to generate and modify the required G-code that the printer reads, utilizing the “Generate G-code” button. A time estimation for each generated project is provided at the bottom left of the tab. The code depends on the user-selected settings and can vary significantly based on the situation. Users can add or remove commands as needed, particularly since the Marlin firmware offers a variety of commands that can be utilized, though most are not useful for the spray process [8]. The generated G-code can be saved, using the “Save G-code to File” button and either be loaded, using the “Load G-code” button or used for offline spraying with a SD card, plugged into the printer.

The third and final tab of the program, labeled “Machine Control” and depicted in Figure S3b, facilitates user interaction with the setup via serial communication. Users can establish a connection to the printer by pressing the “Connect” button when the device is connected to a USB port. Notably, the default baud rate is set to 250000 by the MRiscoC firmware, as opposed to the standard 115200. The “Refresh” button updates the list of available serial ports, enabling users to locate the printer if it was connected after the program was launched. Users can receive information from the printer and send commands via the large command window, using either the “Enter” key on the keyboard or the “Send” button on the bottom right. The “Clear” button erases all content currently displayed in the command window. This tab also includes quick functions, used to move and home the printer’s axes, set the temperature or control various functions like the compressed air and the syringe pump.

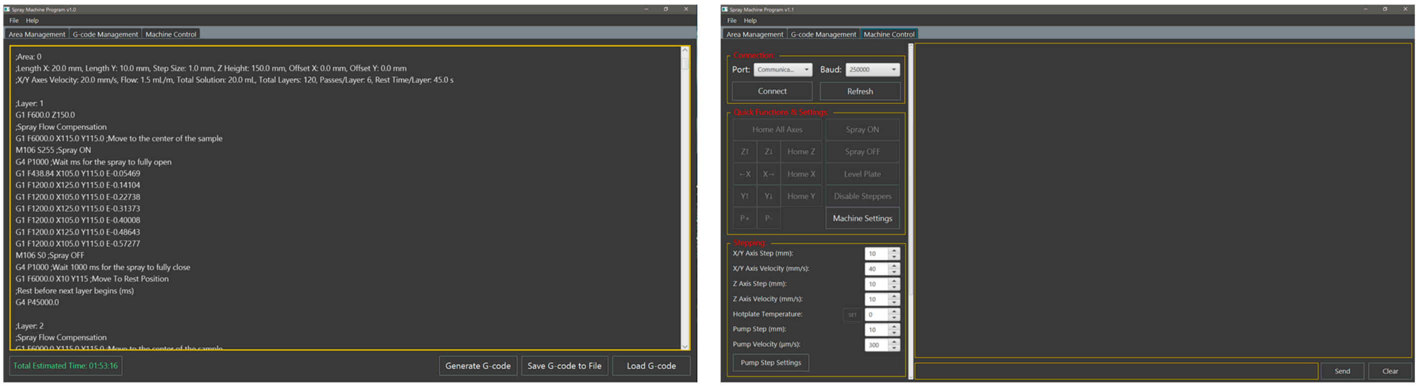

(a) (b)

Figure S3. (**a**) The “G-code Management” tab with a generated example; (**b**) The “Machine Control” tab, used to communicate directly with the setup.

In the “45° Angled Lines” pattern, which was not used for the experimental section, the spray gun begins at the top left corner of the substrate, just like in the “Straight Lines” pattern. It makes a small forward step along the X-axis, then moves diagonally at a 45-degree angle until it reaches an equivalent distance on the Y-axis. The pattern then reverses, stepping forward along the Y-axis and moving diagonally to reach the X-axis at 45 degrees. This alternating pattern continues until the substrate is fully covered. Finally, similarly to the “Straight Lines” pattern, the “45° Angled Lines” pattern features evenly spaced lines. An example of this movement pattern can be observed in Figure S4. The final pattern, “Stationary Center”, involves no movement. In this mode, the spray gun is placed at the center of the substrate and remains stationary, spraying for a specified amount of time. This mode is useful for relatively small substrates

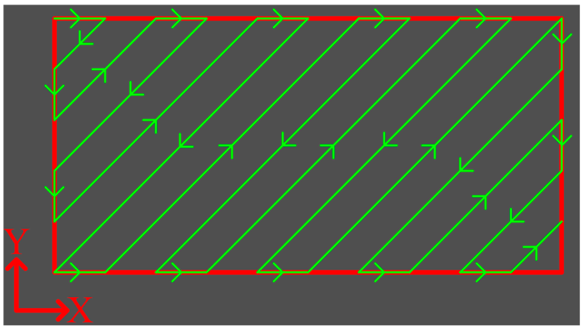

Figure S4. Movement pattern example for the “45° Angled Lines” pattern.

Additional options available in the "Advanced Options" window, as shown in Figures S5a and S5b, but not discussed in the main article or used in the experimental section, include the "Disable Rest Position – Continuous Flow Mode" option, which removes the waiting time between each layer, enabling continuous spray operation. Another option is the "Direction for Straight and Centered Lines," which allows users to change the dominant spray axis from X to Y by selecting "Vertical" instead of "Horizontal", as shown in Figures S5c and S5d for each respective pattern. The "Sample Processing" option determines how multiple spray areas are handled, offering two modes: "Individually" and "In Series". In the "Individually" mode, each sample is fully processed before moving to the next, while in the "In Series" mode, one layer is sprayed on all samples before moving to the rest position, and the process continues layer by layer until all samples are completed. The "In Series" option significantly reduces processing time by allowing solvent evaporation across all samples simultaneously, though it may introduce interference between spray processes among the samples.

The "Automatic" mode, part of the "Area Stepping & Offsets" section, employs an algorithm to calculate the step size based on the substrate dimensions and is further divided into two sub-options: "Full Automatic" and "Semi-Automatic". Both modes use Equations S1 and S2 to determine the number of lines, which is always an integer (natural) number, with the step applied along the non-dominant spray axis. In "Full Automatic" mode, the step and search range are determined based on the sample dimensions and parameters listed in Table S4. In contrast, "Semi-Automatic" mode allows users to define their own search range and step size using the "Range" and "Step" options in the interface. If "Full Automatic" mode fails to find an appropriate number of lines, an offset is applied, as indicated in the corresponding equations, to ensure a correct calculation. These offsets can also be manually adjusted in "Full Manual" mode if users wish to apply specific offsets to the area, using the "Manual X Area Offset (mm)" and "Manual Y Area Offset (mm)" options.

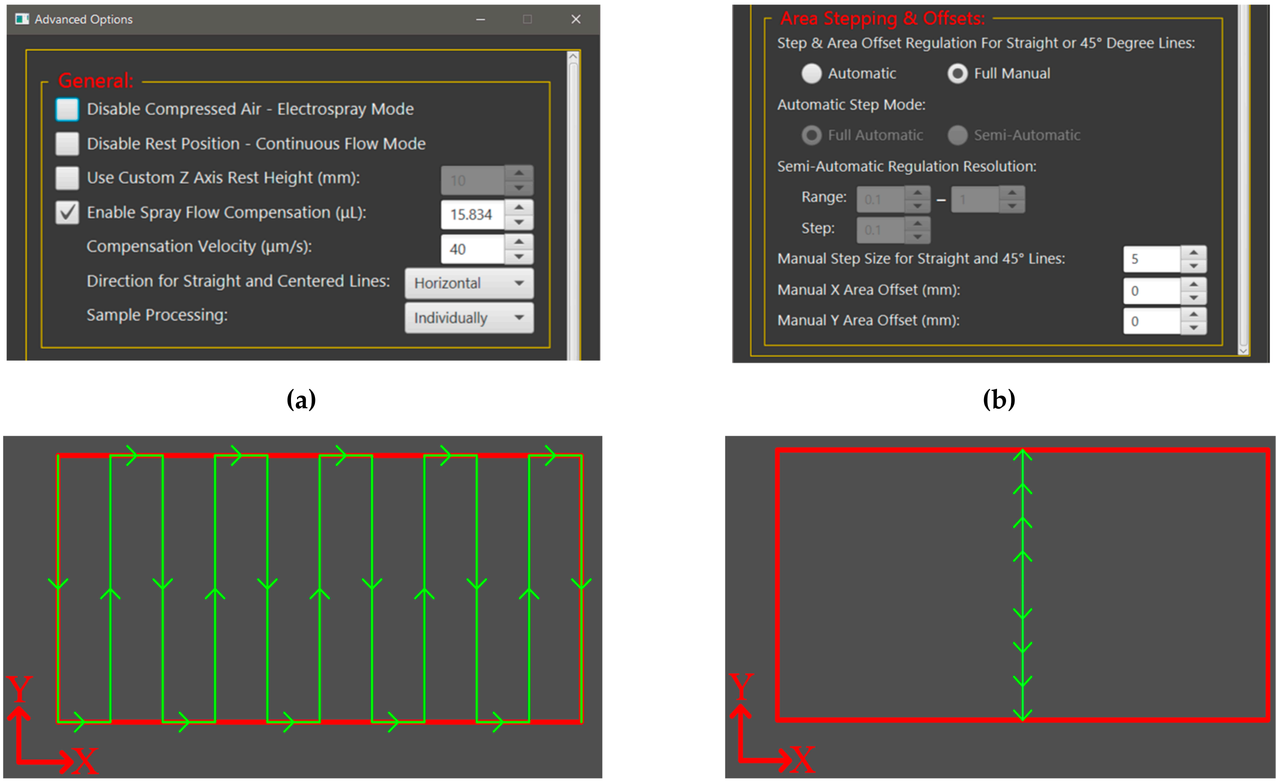

(a) (b) (c) (d)

Figure S5. The "Advanced Options" window (**a**) "General" options; (**b**) "Area Stepping & Offsets" options; and Vertical operation for **(c)** The "Straight Lines" pattern; **(d)** The Centered Line(s) pattern.

$$\text{Line Number(SL)} = (((\text{Non-dominant spray axis substrate length} + \text{Offset Step})) / \text{Step Size}) + 1 \quad \text{(S1)}$$

$$\text{Line Number(45)} = (\text{Substrate X length} + \text{Substrate Y length} + \text{Offset Step} - \text{Step Size}) / \text{Step Size} \quad \text{(S2)}$$

Table S4. The "Full Automatic" searching step algorithm.

Scheme	Start Search Step	End Search Step	Step Size
<10	0.99	0.01	
≥10 and <100	1	10	0.01
100	1	20	

References

1. Samokhin, A. S. "Syringe pump created using 3D printing technology and arduino platform." *Journal of Analytical Chemistry* 75 (2020): 416-421.
2. Pusch, Kira, Thomas J. Hinton, and Adam W. Feinberg. "Large volume syringe pump extruder for desktop 3D printers." *HardwareX* 3 (2018): 49-61.
3. Pearce, Joshua M., N. C. Anzalone, and C. L. Heldt. "Open-source wax RepRap 3-D printer for rapid prototyping paper-based microfluidics." *Journal of laboratory automation* 21.4 (2016): 510-516.

4. KFTechnology. Available online: https://www.kftechnology.it (Accessed 15.09.2024).
5. World Precision Instrument. Available online: https://www.wpi-europe.com (Accessed 15.09.2024).
6. Ossila. Available online: https://www.ossila.com (Accessed 15.09.2024).
7. Drifton official store. Available online: https://www.drifton.eu (Accessed 15.09.2024).
8. Marlin official. Available online: https://marlinfw.org/meta/gcode/ (Accessed 15.09.2024)

Disclaimer/Publisher's Note: The statements, opinions and data contained in all publications are solely those of the individual author(s) and contributor(s) and not of MDPI and/or the editor(s). MDPI and/or the editor(s) disclaim responsibility for any injury to people or property resulting from any ideas, methods, instructions or products referred to in the content.
